# Structure visibility and online adaptation suitability of a new high-performance ring-gantry cone-beam computed tomography imaging system in thoracic radiotherapy

**DOI:** 10.1016/j.phro.2026.100984

**Published:** 2026-04-28

**Authors:** Agustinus J.A.J. van de Schoot, Joan J. Penninkhof, Britt Kunnen, Kimm P. Fremeijer, Elise M. Nicolai – Koornneef, Kirsten Offereins - van Harten, Judith H. Sluijter, Nienke D. Sijtsema, Marjan van de Pol, Raymond de Boer, Maarten L.P. Dirkx, Steven F. Petit

**Affiliations:** Department of Radiotherapy, Erasmus MC Cancer Institute, University Medical Center Rotterdam, PO Box 2040, 3000 CA Rotterdam, the Netherlands

**Keywords:** Cone-beam computed tomography, Image-guided radiotherapy, Thoracic radiotherapy, Lung cancer, Breast cancer, Adaptive radiotherapy, HyperSight CBCT

## Abstract

**Background and purpose:**

A recently introduced high-performance ring-gantry cone-beam computed tomography (CBCT_H_) imaging system promises superior image quality compared with conventional ring-gantry CBCT imaging (CBCT_C_), however, it incurs additional costs. Our aim was to determine the added value of CBCT_H_ compared with CBCT_C_ in thoracic radiotherapy by quantifying structure visibility and online adaptive radiotherapy (oART) suitability.

**Materials and methods:**

Twenty lung cancer patients treated in free-breathing and twenty left-sided breast cancer patients treated in deep-inspiration breath-hold were included in this prospective clinical study. Three CBCT imaging pairs per patient, consisting of CBCT_H_ and CBCT_C_ acquired on consecutive days, were included. Four observers independently scored all 240 CBCT datasets on structure visibility and oART suitability (1–5 scale). The effect of respiratory motion management on image quality enhancement was determined by comparing cohort-specific score differences.

**Results:**

In total, 4440 structure visibility and 960 oART suitability scores were collected. Patient-averaged scores favored CBCT_H_ in 82% (100%) of the cases in the lung (breast) cohort. Compared with CBCT_C_, CBCT_H_-based scores increased significantly (p < 0.05) in both cohorts for structure visibility (lung: 2.8 to 3.1; breast: 3.1 to 3.8) and oART suitability (lung: 2.6 to 2.9; breast: 3.1 to 3.9). Structure visibility (oART suitability) score differences increased with deep-inspiration breath-hold (p < 0.001) compared with free-breathing, by on average 0.38 (0.39).

**Conclusion:**

In thoracic radiotherapy, CBCT_H_ yielded statistically significant improvements in structure visibility and oART suitability where the largest improvements were obtained for CBCT_H_ in deep-inspiration breath-hold. These results can contribute to informed decision-making regarding cost-effectiveness of CBCT_H_.

## Highlights

• HyperSight imaging improves thoracic structure visibility, based on 4440 scores.
• Structure visibility on HyperSight images scored on average 3.4 on a 1–5 scale.
• Breath-hold imaging further enhances structure visibility on HyperSight images.
• Thoracic structure visibility on HyperSight images allows adaptive treatments.
• HyperSight image quality gains enable informed decisions on cost-effectiveness.

## Graphical abstract

**Figure:**
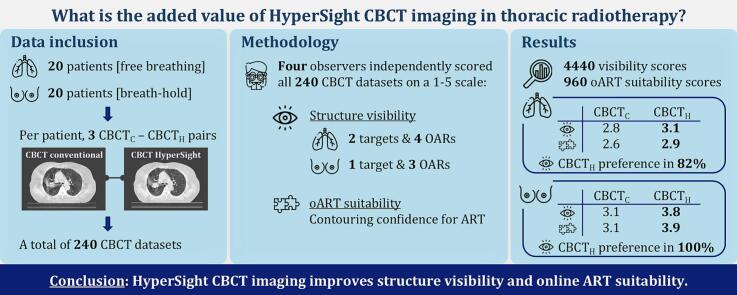

## 1 Introduction

Radiotherapy aims to deliver the prescribed dose to the target volume while sparing surrounding healthy tissue. Image-guided radiotherapy using cone-beam computed tomography (CBCT) is widely used for accurate target volume localization, thereby minimizing patient positioning uncertainties. Since both inter- and intra-fractional anatomical changes may occur during the course of treatment, plan adaptation during the course of treatment can improve target coverage and organ-at-risk (OAR) sparing. Although adaptive radiotherapy (ART) can be performed using different strategies [1], [2], [3], online ART (oART) using CBCT imaging is increasingly applied for various tumor sites, mainly in the pelvic region [4], [5], [6], [7], [8]. However, the implementation of CBCT-based oART in the thoracic region is still rather unexplored.

The clinical implementation of CBCT-based oART demands high-quality CBCT imaging, preferably comparable to fan-beam CT imaging, to enable accurate target and OAR structure definition and dose calculation. Currently, the main challenges for high-quality CBCT imaging are soft tissue differentiation, especially in areas with subtle density differences, and artifact susceptibility. High-quality CBCT imaging is even more challenging in the thoracic region due to respiratory motion, while the suitability of a specific motion management strategy in thoracic cancer radiotherapy mainly relies on the tumor site. The effect of respiratory motion can be reduced using a breath-hold technique, as commonly applied for left-sided breast cancer patients [9], [10]. However, the application of a breath-hold technique in locally advanced stage lung cancer radiotherapy is more challenging and not always feasible due to the patient’s condition, and therefore not yet routinely used [11]. Consequently, high-quality CBCT imaging is, independent of the motion management strategy applied, essential for CBCT-based oART in the thoracic region.

A recently introduced high-performance ring-gantry CBCT imaging system, HyperSight CBCT (Varian Medical Systems, Siemens Healthineers, Palo Alto, CA, USA), offers accelerated acquisitions using a large X-ray detector and advanced reconstructions based on iterative algorithms, resulting in CBCT image quality enhancements [12], [13], [14], [15], [16]. Additionally, the system offers improved Hounsfield unit (HU) accuracy and a larger field-of-view enabling accurate dose calculations [17], [18]. In thoracic cancer radiotherapy, the accelerated CBCT acquisition shortens the required breath-hold time for patients, thereby facilitating breath-hold imaging [10], and reduces motion artefacts during free-breathing imaging [14]. However, the high-performance ring-gantry CBCT imaging system incurs additional costs while the translation of these advancements into clinical or operational benefits remains to be demonstrated [19].

This study determines the added value of high-performance ring-gantry CBCT imaging compared with conventional ring-gantry CBCT imaging in thoracic cancer radiotherapy by quantifying target and OAR structure visibility and suitability for online adaptive radiotherapy in lung cancer and breast cancer patients. Furthermore, the impact of motion management strategy on image quality enhancement was assessed.

## 2 Materials & methods

### 2.1 Patients and treatment

Forty patients, including twenty consecutive lung cancer patients and twenty consecutive left-sided breast cancer patients, treated with non-adaptive image-guided radiotherapy were included in this study, after providing informed consent (MEC-2022–0815). Patients eligible for inclusion received intermediate- or long-course fractionated radiotherapy according to our standard clinical practice between March and November 2023 on an Ethos therapy system (Varian Medical Systems) equipped with either conventional CBCT imaging (CBCT_C_) or the high-performance HyperSight CBCT imaging (CBCT_H_).

For lung cancer patients, CBCT imaging and fractionated dose delivery were executed in free-breathing (FB). The median age in the lung cohort was 65 years (range: 36–85). Left-sided breast cancer patients with a median age of 52 years (range: 41–71) were imaged and received fractionated dose delivery in deep-inspiration breath-hold (DIBH). The mean breast CTV was 772.4 cm^3^ (range: 289.1–1678.8). At the treatment machine, the voluntary DIBH was monitored using surface guidance (IDENTIFY, Varian Medical Systems) and real-time feedback on the DIBH level was provided to patients using a visual coaching device. Supplementary Tables S1 and S2 show cohort-specific patient and treatment characteristics.

### 2.2 Patient imaging

All imaging included in this study was acquired as part of our standard clinical practice: 4D-CT imaging (lung cancer) or DIBH-based 3D-CT imaging (breast cancer) for radiotherapy treatment planning purposes and 3D-CBCT imaging for daily pre-fraction position verification. In our institute, lung cancer patients with a respiratory-induced tumor motion amplitude above 10 mm or a respiratory frequency below eight breaths per minute, as measured on 4D-CT imaging, were not eligible for treatment on a CBCT_H_-equipped Ethos therapy system. This exclusion was to avoid a possible mismatch in average tumor position larger than 1.0 mm compared to the 50% exhale phase of 4D-CT used for treatment planning [17], [20]. Consequently, patients with tumor motion amplitudes above 10 mm or respiratory frequencies below eight breaths per minute were not included.

Included patients were treated on an Ethos therapy system equipped with CBCT_H_ and on a CBCT_C_-equipped Ethos therapy system at least once a week, resulting in multiple pairs of CBCT_H_ and CBCT_C_ acquired on consecutive days. Three CBCT pairs per patient were randomly selected to equalize the number of datasets per patient, resulting in 240 included CBCT datasets.

All CBCT imaging was acquired using standard lung cancer or breast cancer protocols, involving fixed exposure and a standard reconstruction technique for CBCT_C_. In contrast, the CBCT_H_ protocols included auto-adjusted exposure and an advanced, iterative reconstruction technique. Table 1 shows image acquisition and reconstruction details.

**Table 1:** CBCT imaging acquisition and reconstruction details.

	Lung cohort		Breast cohort	
	CBCT_C_	CBCT_H_	CBCT_C_	CBCT_H_
Ethos acquisition mode	*Thorax*	*Thorax*	*Breast*	*Breast*
Energy [kVp]	*125*	*125*	*125*	*125*
Exposure [mAs]	*300.65*	*mean: 283.20* *(range: 54.00 – 345.00)*	*49.10*	*mean: 43.40* *(range: 33.00 – 54.00)*
Scan time [s]	*30.8*	*5.9*	*16.6*	*5.9*
Reconstruction algorithm	*Standard (FDK)*	*iCBCT Acuros*	*Standard (FDK)*	*iCBCT Acuros*
In-plane resolution [mm^2^]	*0.96*	*1.05*	*0.96*	*1.05*
Slice thickness [mm]	*2.0*	*2.0*	*2.0*	*2.0*
Reconstruction diameter [mm]	*492*	*538*	*492*	*538*

*Abbreviations*: CBCT_C_, conventional CBCT; CBCT_H_, HyperSight CBCT; FDK, Feldkamp-Davis-Kress; iCBCT, iterative CBCT.

### 2.3 Structure visibility and online ART suitability

**Fig. 1:**
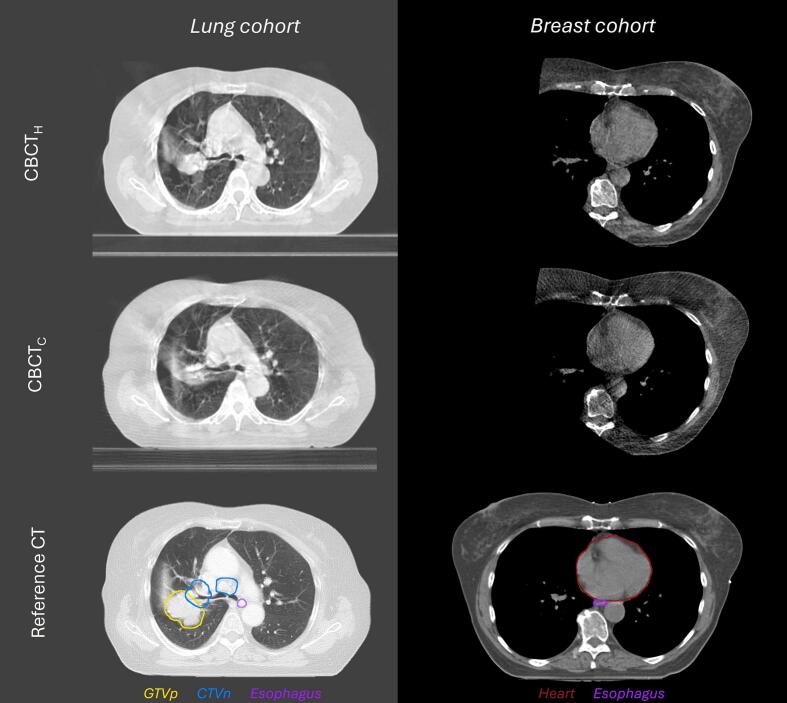
Examples of CBCT_H_ (first row), CBCT_C_ (second row) and reference CT with reference structures (third row) at corresponding axial slices for the lung cohort (first column) and breast cohort (second column).

All 240 CBCT datasets were pseudo-anonymized, blinded, and evaluated individually by two experienced radiation technologists (RTT) out of a pool of three and two experienced radiation oncologists (RO) in an arbitrary order. To facilitate an efficient and systematic evaluation process, a workflow was created in MIM Maestro (MIM Software Inc., version 7.1.6). The CBCT image and CT image used for radiotherapy treatment planning (lung cancer: 50% exhale phase of 4D-CT; breast cancer: DIBH-CT) were rigidly aligned on bony anatomy and shown next to each other to facilitate comparison of corresponding axial slices, including visualization of target and OAR delineations on the reference CT. All CBCT images in the breast cohort were masked on the contralateral side to conceal machine-specific field-of-view details, Fig. 1.

The target and OAR structures considered relevant for this study were the primary GTV (GTVp), nodal CTV (CTVn), esophagus, heart, aortic arch, and brachial plexus for lung cancer patients and the tumor bed, esophagus, heart, and aortic arch for breast cancer patients. The brachial plexus structure was not present on CBCT imaging of breast cancer patients due to the isocenter position in combination with the limited CBCT field-of-view. Structure visibility on the CBCT images was graded using a 1–5 scale [12]:

1) Not or barely visible, with insufficient quality for delineation.
2) Poorly visible and of unsatisfactory quality for delineation.
3) Sufficiently visible and of fair quality for delineation.
4) Well visible and of good quality for delineation.
5) Clearly visible and of excellent quality for delineation.

Each observer independently scored each structure on every CBCT image. In addition to structure scores, each observer also provided an oART suitability score per CBCT image based on the overall image quality. This oART suitability score was defined as the observer confidence for CBCT-based contour definition during online adaptive radiotherapy and was provided on a 1–5 scale, ranging from ‘completely not confident’ to ‘completely confident’ respectively.

### 2.4 Statistical analysis

Structure visibility score differences were calculated for each patient, structure and observer by subtracting the CBCT_C_ visibility score from the CBCT_H_ visibility score per CBCT image pair. Similarly, oART suitability score differences were calculated per CBCT image pair for each patient and observer. Patient-averaged structure visibility score differences per structure and patient-averaged oART suitability score differences were derived by averaging over the three CBCT pairs and four observers. Improvements in patient-averaged structure visibility and oART suitability scores for CBCT_H_ compared with CBCT_C_ were tested for significance (Wilcoxon signed-rank test). Average differences in structure visibility and oART suitability scores were also calculated per observer group (i.e., RO and RTT) and group-specific score distributions were compared (Mann-Whitney *U* test) to verify whether both groups experience similar differences between CBCT_H_ and CBCT_C_.

### 2.5 Respiratory motion management

The effect of respiratory motion on CBCT image quality enhancement was investigated by calculating the Pearson correlation coefficients between patient-specific tumor motion amplitudes obtained during 4D-CT imaging and average score differences for lung cancer patients. The visibility of the heart, esophagus and aortic arch as well as oART suitability was scored in both the lung and breast cohort. To determine the effect of the applied respiratory motion management technique, FB for lung cancer patients and DIBH for breast cancer patients, on image quality enhancement, average differences in structure visibility and oART suitability scores per cohort were compared (Mann-Whitney *U* test).

## 3 Results

**Fig. 2:**
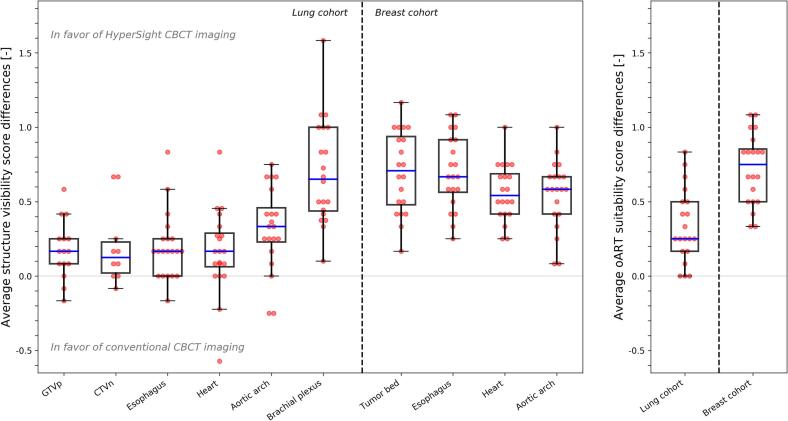
Average differences in structure visibility score (left) and average differences in oART suitability score (right) for lung cancer patients (left from vertical dotted line) and breast cancer patients (right from vertical dotted line), defined as CBCT_H_ minus CBCT_C_. Boxes represent upper and lower quartiles (IQR), with the blue band representing the median. Whiskers show the highest (lowest) value within 1.5*IQR of the upper (lower) quartile. Dots denote individual patients. (For interpretation of the references to colour in this figure legend, the reader is referred to the web version of this article.)

**Fig. 3:**
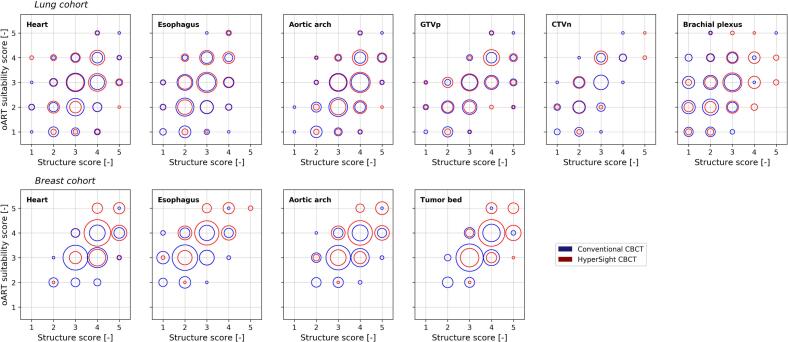
Bubble plots showing the relation between structure visibility scores and oART suitability scores for CBCT_C_ imaging (blue) and CBCT_H_ imaging (red) for each structure in the lung cohort (first row) and the breast cohort (second row). The bubble size corresponds to the frequency of collected structure visibility score and oART suitability score combinations, with larger bubbles representing higher score frequencies. (For interpretation of the references to colour in this figure legend, the reader is referred to the web version of this article.)

A total of 4440 structure visibility scores, of which 2520 in the lung cohort and 1920 in the breast cohort, and 960 oART suitability scores were collected. Within the lung cohort, GTVp and CTVn were present and subsequently scored for visibility in 15 and 10 patients, respectively. Neither GTVp nor CTVn was scored in two patients who received postoperative radiotherapy. Patient-averaged structure visibility scores are shown in Fig. 2 and demonstrated CBCT_H_ favorability in 82% of the cases in the lung cohort and 100% in the breast cohort. In the lung cohort, the mean visibility scores on CBCT_H_ compared with CBCT_C_ increased significantly (p < 0.05) from 3.1 to 3.3 for the GTVp, 2.5 to 2.7 for the CTVn, 3.1 to 3.3 for the heart, 2.6 to 2.8 for the esophagus, 3.3 to 3.6 for the aortic arch, and 2.1 to 2.8 for the brachial plexus. In the breast cohort, the mean visibility scores on CBCT_H_ compared with CBCT_C_ increased significantly (p < 0.001) from 3.3 to 4.0 for the tumor bed, 3.5 to 4.1 for the heart, 2.3 to 3.0 for the esophagus, and 3.4 to 3.9 for the aortic arch. The mean oART suitability score on CBCT_H_ compared with CBCT_C_ increased from 2.6 to 2.9 in the lung cohort and from 3.1 to 3.9 in the breast cohort. Fig. 3 shows the relation between visibility scores and oART suitability scores for individual structures per cohort.

**Fig. 4:**
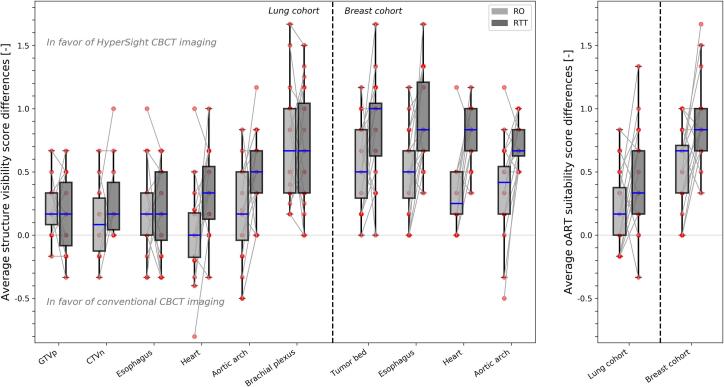
Average differences in structure visibility score (left) and average differences in oART suitability score (right) for lung cancer patients (left from vertical dotted line) and breast cancer patients (right from vertical dotted line), defined as CBCT_H_ minus CBCT_C_, divided between observer groups (i.e., RO and RTT). Boxes represent upper and lower quartiles (IQR), with the blue band representing the median. Whiskers show the highest (lowest) value within 1.5*IQR of the upper (lower) quartile. Dots denote individual patients, and grey lines connect corresponding patients. (For interpretation of the references to colour in this figure legend, the reader is referred to the web version of this article.)

Patient-averaged structure visibility score differences and oART suitability score differences between CBCT_H_ and CBCT_C_ per observer group are shown in Fig. 4. Compared with the RO group, visibility score differences in the lung cohort for the RTT group significantly increased on average from 0.02 to 0.32 (p < 0.01) for the heart and from 0.16 to 0.48 (p < 0.05) for the aortic arch, while for other structures both observer groups scored similar differences between CBCT_H_ and CBCT_C_. In the breast cohort, the RTT group scored significantly larger visibility differences compared with the RO group for the heart (0.81 vs 0.32; p < 0.001), aortic arch (0.73 vs 0.38; p < 0.001), esophagus (0.90 vs 0.50; p < 0.01), and tumor bed (0.85 vs 0.55; p < 0.05). The oART suitability score differences were on average larger for the RTT group in the lung cohort (0.45 vs 0.22; p < 0.05) and the breast cohort (0.90 vs 0.54; p < 0.01).

The mean tumor motion amplitude of lung cancer patients was 2.9 mm (range: 0.2 – 8.9). The correlation coefficient between tumor motion amplitudes and average score differences was −0.06 for the GTVp, −0.39 for the CTVn, 0.23 for the heart, −0.10 for the esophagus, −0.06 for the aortic arch, −0.03 for the brachial plexus, and −0.43 for oART suitability, indicating decreased average score differences for increasing motion amplitudes. Structure visibility score differences in the DIBH-based breast cohort were significantly higher compared with the FB-based results in the lung cohort, by on average 0.40 for the heart (p < 0.001), 0.50 for the esophagus (p < 0.001) and 0.23 for the aortic arch (p < 0.05). Also, oART suitability score differences in the DIBH-based breast cohort were significantly higher compared with the FB-based differences in the lung cohort, by on average 0.39 (p < 0.001).

## 4 Discussion

A recently introduced high-performance ring-gantry CBCT imaging system demonstrated superior CBCT image quality for various anatomical sites [12], [14], but incurs additional costs. Therefore, this study determined the added value of the high-performance ring-gantry CBCT imaging compared to conventional ring-gantry CBCT imaging for thoracic radiotherapy in terms of target and OAR structure visibility as well as oART suitability. For all structures, we demonstrated a significantly increased CBCT_H_-based structure visibility and oART suitability compared with CBCT_C_ for both FB-based and DIBH-based image acquisition. The increase in structure visibility and oART suitability for CBCT_H_ compared with CBCT_C_ was significantly larger when CBCT imaging was performed in combination with a DIBH technique compared with FB-based CBCT imaging.

Given the scoring scale of 1–5, structures on CBCT_H_ were scored either sufficiently visible in the lung cohort or well visible in the breast cohort. Additionally, CBCT_H_-based mean oART suitability scores represented either a fair level of confidence in the lung cohort or a good level of confidence in the breast cohort for CBCT_H_-based delineation during an adaptive workflow. Improvements in oART suitability for CBCT_H_ are consistent with increased CBCT_H_-based structure visibility scores. Considering all results, we conclude that DIBH-based CBCT_H_ is suitable for target and OAR delineation in breast radiotherapy and contributes to adaptive treatments, while structure delineation on FB-based CBCT_H_ in lung cancer remains challenging. Since respiratory motion not only challenges accurate delineation but may also induce HU inaccuracies in CBCT_H_, hampering accurate dose calculations [17], the clinical applicability of FB-based oART in lung cancer patients currently appears limited. To determine the feasibility of DIBH-based oART in lung cancer patients, additional investigation is needed for, among other things, DIBH-based CBCT_H_ image quality and DIBH reproducibility.

Visibility scores differed clearly between observer groups for the heart and aortic arch in the lung cohort and all structures in the breast cohort. For those structures, RTTs assigned higher scores to CBCT_H_ than ROs. Although the limited number of observers may have affected these differences and they could potentially be explained by the fact that our RTTs are used to interpret CBCT imaging on a daily basis in clinical practice, while ROs are primarily used to interpret imaging modalities such as CT and MRI. Moreover, our RTTs are used to perform CBCT_H_-based oART for various indications using an RTT-led workflow.

Our results on structure visibility improvements on CBCT_H_ agree with previous phantom-based studies [13], [15], [16], [18] as well as patient-based studies in the pelvic and thoracic region [12], [14] on CBCT_H_ image quality enhancement. Although structure visibility improvements on CBCT_H_ in lung cancer patients were relatively small, our results indicate CBCT_H_ image quality enhancement for non-static anatomical imaging combined with patient-specific, irregular respiratory motion. Larger improvements in CBCT_H_-based structure visibility were found in breast cancer patients with an average visibility score of 3.8, however still inferior compared with reported CBCT_H_-based average structure visibility score of 4.5 in prostate cancer patients [12].

A major study limitation was the inclusion of only FB-based CBCT imaging in the lung cohort and only DIBH-based CBCT imaging in the breast cohort. Structure visibility and oART suitability improvements were therefore not quantified in DIBH-based CBCT imaging for lung cancer patients and FB-based CBCT imaging for breast cancer patients. The study would have been strengthened by incorporating both DIBH-based and FB-based imaging data for all patient groups to eliminate cohort-specific consequences. Unfortunately, the necessary data for this study extension is not available since DIBH-based lung cancer treatments are not performed on Ethos therapy systems in our institute. The breast cohort results still indicate CBCT_H_ image quality enhancements compared with CBCT_C_ using DIBH.

Another factor limiting the comparison between CBCT_C_ and CBCT_H_ was the inclusion of imaging data obtained using standard clinical protocols. All CBCT imaging in this study was acquired using standard exposure for CBCT_C_, while the exposure for CBCT_H_ was automatically adjusted by the system based on patient size. In most cases, the exposure used for CBCT_H_ acquisition was lower than the corresponding CBCT_C_ exposure. Nevertheless, CBCT_H_ image quality enhancement was observed.

The use of standard clinical protocols also resulted in CBCT_H_ imaging based on iterative reconstructions. Although the use of the iterative reconstruction algorithm is prone to respiratory artifacts, FB-based CBCT_H_ images were reconstructed using this advanced algorithm to increase HU accuracy for CBCT_H_-based dose calculations. Consequently, CBCT_H_ scores in the lung cohort may have been influenced negatively by respiratory-induced reconstruction artifacts and the use of a non-iterative reconstruction algorithm could further increase CBCT_H_-based structure visibility. It would have been interesting to relate image quality enhancements to differences in hardware, image acquisition and image reconstruction, however within the current study this was not possible due to technical and ethical reasons.

Given the acquisition time of 5.9 s for CBCT_H_, the image quality was subject to the patient-specific respiratory motion during FB-based image acquisition [17], [20]. In our clinical practice, patients with a large respiratory amplitude or a low respiratory rate are excluded from CBCT_H_-based treatment to avoid a possible mismatch in average tumor position compared to reference imaging, and consequently not represented in this study. Nevertheless, patients with various respiratory amplitudes were included in the lung cohort. The impact of these patient-specific motion amplitudes were however limited, indicated by the weak correlation found between tumor motion amplitudes and structure visibility as well as oART suitability scores.

In conclusion, we evaluated the added clinical value of the high-performance ring-gantry CBCT imaging system compared with conventional ring-gantry CBCT imaging in thoracic cancer radiotherapy in terms of target and OAR structure visibility as well as online ART suitability for different respiratory motion management strategies. High-performance CBCT imaging acquired in free-breathing yielded moderate, but statistically significant improvements in structure visibility and online ART suitability. Larger, statistically significant improvements in both structure visibility and online ART suitability were obtained for high-performance CBCT image acquisition in DIBH. In combination with our previous findings on the added value of CBCT_H_ imaging for prostate cancer patients [12], these results contribute to informed decision-making regarding cost-effectiveness of this high-performance CBCT imaging system.

## CRediT authorship contribution statement

**Agustinus J.A.J. van de Schoot:** Writing – review & editing, Writing – original draft, Visualization, Validation, Supervision, Software, Project administration, Methodology, Investigation, Formal analysis, Conceptualization. **Joan J. Penninkhof:** Writing – review & editing, Visualization, Supervision, Methodology, Formal analysis, Conceptualization. **Britt Kunnen:** Writing – review & editing, Validation, Methodology, Investigation, Conceptualization. **Kimm P. Fremeijer:** Writing – review & editing, Validation, Methodology, Investigation. **Elise M. Nicolai – Koornneef:** Writing – review & editing, Validation, Methodology, Investigation. **Kirsten Offereins - van Harten:** Writing – review & editing, Validation, Methodology, Investigation. **Judith H. Sluijter:** Writing – review & editing, Validation, Methodology. **Nienke D. Sijtsema:** Writing – review & editing, Validation, Methodology. **Marjan van de Pol:** Writing – review & editing, Validation, Methodology, Investigation. **Raymond de Boer:** Writing – review & editing, Validation, Methodology, Investigation. **Maarten L.P. Dirkx:** Writing – review & editing, Validation, Supervision, Methodology, Formal analysis, Conceptualization. **Steven F. Petit:** Writing – review & editing, Validation, Supervision, Software, Methodology, Formal analysis, Conceptualization.

## Declaration of competing interest

The authors declare the following financial interests/personal relationships which may be considered as potential competing interests: Erasmus MC Cancer Institute has research collaborations with Elekta AB (Stockholm, Sweden), Accuray Inc. (Sunnyvale, CA, USA) and Varian, a Siemens Healthineers Company (Palo Alto, CA, USA). Varian was not involved in this study and had no role in study design, data collection and analysis, and decisions on preparation the manuscript. None of the authors has any affiliation with Varian. There are no other conflicts of interest to declare from all authors. No funding was received for this study.

## Appendix A Supplementary data

Supplementary Data 1 Patient and treatment characteristics.
