# Supplementary material for: Structure visibility and online adaptation suitability of a new high-performance ring-gantry cone-beam computed tomography imaging system in thoracic radiotherapy
Source: Phys Imaging Radiat Oncol. 2026 Apr 28;39:100984. doi: 10.1016/j.phro.2026.100984 (PMC13147364; doi:10.1016/j.phro.2026.100984)
Supplement: Supplementary Data 1 — Patient and treatment characteristics. [file mmc1.pdf]

Supplementary Material: Patient and treatment characteristics

Supplementary Table S1. *Patient and treatment characteristics included in the lung cohort.*

Patient	Sex	Age [yr]	Tumor stage	Tumor location	Treatment	Fractionation	GTVp [cm^3]	CTVn [cm^3]	Motion amplitude [mm]
1	Female	69	T3N0M0	LUL	CCRT	30 x 2.00 Gy	132.0	23.4	1.0
2	Female	74	cT3N1M1b	RLL / RML	CCRT	33 x 2.00 Gy	114.9	-	1.5
3	Male	51	cT4N0M0	LUL	CCRT	30 x 2.00 Gy	256.1	-	0.2
4	Male	72	cT2N2M0	RML	CCRT	30 x 2.00 Gy	62.7	-	6.2
5	Male	85	T4N0M0	LLL	SCRT	20 x 2.75 Gy	40.4	-	1.6
6	Male	80	T1cN0M0	LUL	SCRT	24 x 2.75 Gy	-	39.1	4.9
7	Male	78	cT1cN2M0	RUL	CCRT	30 x 2.00 Gy	6.3	35.4	8.9
8	Female	57	cT4N2M0	RUL	CCRT	30 x 2.00 Gy	102.0	44.1	1.2
9	Male	58	pT1cN0M0	RUL	PORT	30 x 2.00 Gy	-	-	7.3
10	Male	60	cT3N2M0	RLL	SCRT	20 x 2.75 Gy	21.3	75.7	3.4
11	Male	72	T3N0M0	LUL	CCRT	30 x 2.00 Gy	45.9	-	4.4
12	Male	76	pT3N1M0	RUL	PORT	30 x 2.00 Gy	-	-	0.4
13	Female	48	cT3N0M0	RUL	CCRT	30 x 2.00 Gy	117.7	-	0.2
14	Female	36	cT2N2M1b	RUL	SCRT	20 x 2.75 Gy	100.7	-	2.2
15	Female	64	T2bN2M0	RUL	CCRT	30 x 2.00 Gy	42.6	66.7	4.7
16	Male	61	cT0N2M0	RUL	CCRT	33 x 2.00 Gy	7.6	27.6	1.2
17	Female	65	T2aN2M0	RUL	CCRT	30 x 2.00 Gy	30.5	-	3.4
18	Male	59	T0N2M1b	Mediastinum	RT	20 x 2.75 Gy	-	91.3	1.3
19	Male	67	T4N2M1	LUL	CCRT	30 x 2.00 Gy	725.5	12.3	2.3
20	Female	60	cT4bN2M0	RUL / RML	SGRT	24 x 2.75 Gy	-	74.4	2.1

Abbreviations: RUL, right upper lobe; RML, right middle lobe; RLL, right lower lobe; LUL, left upper lobe; LLL, left lower lobe; CCRT, concurrent chemoradiotherapy; SCRT, sequential chemoradiotherapy; PORT, postoperative radiotherapy; RT, radiotherapy.

Supplementary Table S2. *Patient and treatment characteristics included in the breast cohort.*

Patient	Age [yr]	Tumor stage	Treatment	Fractionation	Breast CTV [cm^3]
1	49	ypT1bN0(i-)(sn)	WBRT	15 x 2.67 Gy	679.9
2	69	pT1cN0 (i-)(sn)	WBRT	15 x 2.67 Gy	571.5
3	41	ypT1miN0(i-)(sn)	WBRT	15 x 2.67 Gy	716.5
4	51	ypT0N0(RISAS)	WBRT	15 x 2.67 Gy	598.7
5	46	ypT1bN0(i+)(sn)	LRT [AXL, SCL, IMN]	15 x 2.67 Gy	768.7
6	48	pT1cN0(i-)(sn)	WBRT	15 x 2.67 Gy	815.2
7	57	ypT1bN0(i-)(sn)	WBRT	20 x 2.67/2.18 Gy [SIB]	504.7
8	49	ypT0N0	LRT [AXL, SCL]	15 x 2.67 Gy	425.2
9	58	pT1cN0(i+)(sn)	LRT [AXL]	15 x 2.67 Gy	1038.9
10	67	pT1cN0(i-)(sn)	WBRT	15 x 2.67 Gy	726.9
11	52	pT1cN1mi(sn)	WBRT	15 x 2.67 Gy	963.7
12	52	ypT0N0(i-)(sn)	WBRT	15 x 2.67 Gy	1230.9
13	69	pT2N0(i-)(sn)	WBRT	15 x 2.67 Gy	926.0
14	65	pTisN0(i-)(sn)	WBRT	15 x 2.67 Gy	847.0
15	50	pT1c(m)N0(i+)(sn)	WBRT	15 x 2.67 Gy	668.8
16	60	pT1aN0(i-)(sn)	WBRT	15 x 2.67 Gy	785.1
17	47	pT1aN0(i-)(sn)	WBRT	15 x 2.67 Gy	289.1
18	48	pT1cN0(i-)(sn)	WBRT	15 x 2.67 Gy	845.7
19	61	pTis	WBRT	15 x 2.67 Gy	1678.8
20	71	pT1bN0(i-)(sn)	WBRT	15 x 2.67 Gy	366.8

Abbreviations: WBRT, whole-breast radiotherapy; LRT, locoregional radiotherapy; AXL, axillary lymph nodes; SCL, supraclavicular lymph nodes; IMN, internal mammary nodes; SIB, simultaneously integrated boost.
